# Evolution of body composition following successful kidney transplantation is strongly influenced by physical activity: results of the CORPOS study

**DOI:** 10.1186/s12882-020-02214-9

**Published:** 2021-01-18

**Authors:** Karine Moreau, Aurélie Desseix, Christine Germain, Pierre Merville, Lionel Couzi, Rodolphe Thiébaut, Philippe Chauveau

**Affiliations:** 1 Renal Transplant Unit, Pellegrin Hospital, Bordeaux, France; 2 AURAD Aquitaine, Gradignan, France; 3 Clinical Epidemiology Unit, Bordeaux University Hospital, Bordeaux, France; 4 CNRS-UMR 5164 immunoConcEpT Bordeaux University, Bordeaux, France; 5 Bordeaux University, Bordeaux, France; 6 INSERM U1219 Bordeaux Population Health, Bordeaux, France

## Abstract

**Background:**

Weight gain (mainly gain of fat mass) occurs quickly after successful kidney transplantation and is associated with metabolic complications (alterations of glycaemic control, hyperlipidaemia). Determinants of weight gain are multifactorial and are mainly related to the transplant procedure itself (glucocorticoid use, increased appetite). In the modern era of transplantation, one challenge is to limit these metabolic alterations by promoting gain of muscle mass rather than fat mass. This prospective study was performed to assess determinants of fat mass, fat-free mass and body cell mass changes after kidney transplantation with a focus on physical activity and nutritional behaviour before and after transplantation.

**Methods:**

Patients were included at the time of listing for deceased donor kidney transplantation. Body composition was determined using dual X-ray absorptiometry and bioimpedance spectroscopy to assess fat mass, fat-free mass and body cell mass (= fat-free mass − extracellular water) at the time of inclusion, 12 months later, and 1, 6, 12 and 24 months after transplantation. Recall dietary data and physical activity level were also collected.

**Results:**

Eighty patients were included between 2007 and 2010. Sixty-five had a complete 24-month follow-up after kidney transplantation. Fat mass, fat-free mass and body cell mass decreased during the waiting period and early after kidney transplantation. The nadirs of body cell mass and fat-free mass occurred at 1 month and the nadir for fat mass occurred at 6 months. Maximum levels of all parameters of body composition were seen at 12 months, after which body cell mass and fat-free mass decreased, while fat mass remained stable. In multivariate analysis, male recipients, higher physical activity level and lower corticosteroid dose were significantly associated with better body cell mass recovery after kidney transplantation.

**Conclusions:**

Lifestyle factors, such as physical activity level, together with low dose of corticosteroids seem to influence body composition evolution following kidney transplantation with recovery of body cell mass. Specific strategies to promote physical activity in kidney transplant recipients should be provided before and after kidney transplantation.

## Background

Kidney transplantation (KT) is the treatment of choice for end-stage renal failure, including in patients who are obese at the time of KT. However, such obese patients are at high risk of complications after KT [1]. Independently of weight at the time of KT, weight gain after KT is a well-known side effect of successful kidney transplantation. It occurs quickly in the first months after transplantation, and is mainly related to an increase in fat mass. During the same period, fat-free mass remains stable or increases slightly [2–4]. The consequences of these body composition changes are not yet clear. They have been reported to be associated with higher prevalence rates of hypertension and diabetes [5] or dyslipidaemia [6], which may explain the elevated risk of death-censored graft loss and death with a functioning graft observed in obese kidney transplant recipients (KTR) [5, 7]. However, Chang et al. described a U-shaped relationship between post-transplant weight changes and survival outcomes, and showed that a moderate (10–19.9%) weight gain during the first year was associated with the best outcome [8]. Increment in body weight after transplantation may reflect normalisation of a pre-existing malnourished state. We demonstrated previously that in French patients waiting for kidney transplantation, body composition was altered despite satisfactory classical nutritional markers [9].

Causes of post-transplant weight gain are multifactorial. Some are non-specific, such as age, sex, genetics, socioeconomic status, dietary habits and pre-transplant obesity. Specific items have also been identified including the disappearance of dietary restrictions, increased appetite (feeling of well-being and glucocorticoids) and decreased physical activity in the first months after transplantation.

This prospective study was performed in patients before and after kidney transplantation to determine changes in whole-body composition and body cell mass (BCM) within the first 2 years after kidney transplantation and to analyse factors leading to these changes with a specific focus on physical activity, nutritional behaviour and pre-transplant whole-body composition evolution.

## Methods

### Study design

**Fig. 1:**
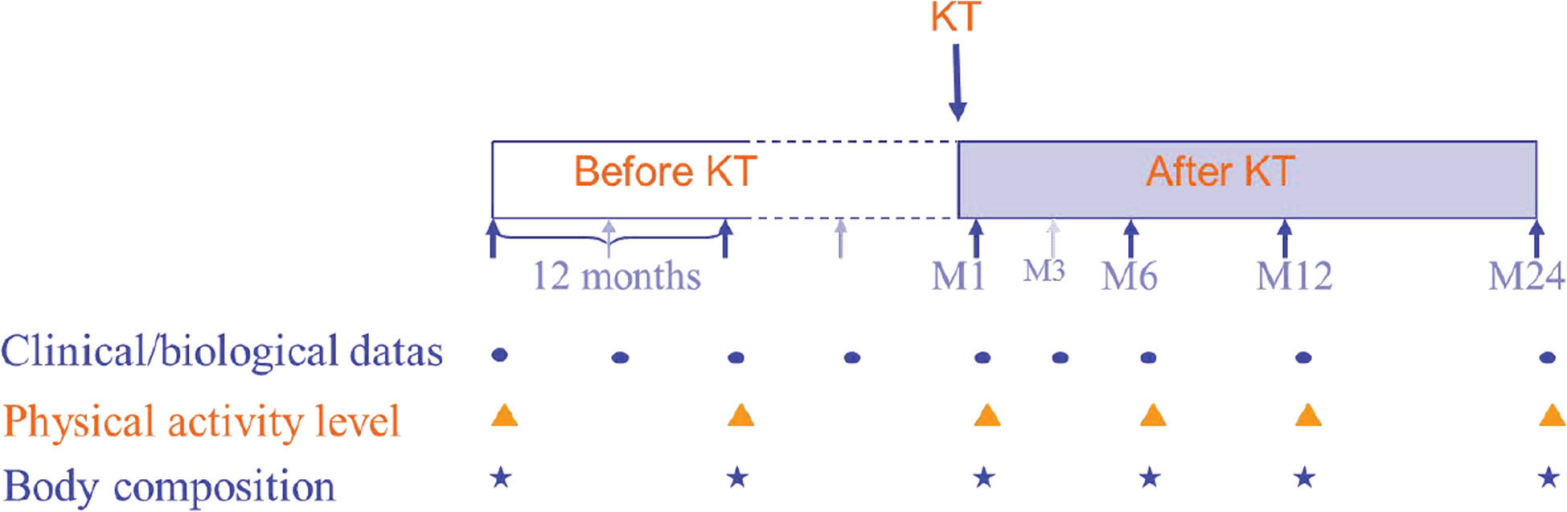
study design. KT = Kidney Transplantation

The CORPOS study (French acronym for “corps” [body] and “os” [bone]) is a prospective longitudinal study in which candidates for first kidney transplantation were tested at the time of listing (baseline), 12 months before kidney transplantation (waiting period) and on months 1, 3, 6, 12 and 24 post-kidney transplantation (Fig. 1). Patient eligibility criteria were described previously [9]. The main inclusion criteria were patients on dialysis aged 18–65 years waiting for first kidney transplantation from a deceased donor. Enrolment was performed between August 2007 and January 2010. KT occurred between 2008 and December 2011. End of follow-up for the last kidney transplant patient occurred in December 2013. The hospital ethics committee approved the study (Comité de Protection des Personnes de Bordeaux A, number 2006/28, June 2006) and all subjects provided informed consent.

At the time of transplantation, the immunosuppressive regimen included induction therapy by either polyclonal anti-thymocyte globulin (in anti-HLA-sensitised patients) or monoclonal anti-CD25 antibody. The maintenance immunosuppressive regimen included calcineurin inhibitors (tacrolimus or cyclosporine in cases of medical intolerance to tacrolimus) and mycophenolate mofetil. Tacrolimus trough levels were maintained between 8 and 10 ng/mL during the first 12 months after transplantation and later decreased to 6–8 ng/mL. Prednisone was stopped on day 7 in non-sensitised patients, except in whose who had received corticosteroid treatment before transplantation.

### Biochemical analysis

All measurements were performed after an overnight fast (before starting dialysis on a midweek dialysis day during the waiting period before KT). Serum total proteins and creatinine were assessed by standard techniques. Albumin was measured using the green bromocresol technique, prealbumin and C-reactive protein using an immunoturbimetric test Dietary recall.

Mean total energy and protein intakes were assessed from the average of 3-day food records. Patients wrote down everything eaten, including food portion sizes, followed by an interview with a specialised dietician to ensure accuracy of reporting. Calculations were performed with a nutrient analysis program (Bilnut 4.0; SCDA Nutrisoft, Le Hallier, Cerelles, France).

### Whole-body composition analysis

Anthropometry: patients wearing light clothing and no shoes were weighted twice to the nearest 0.1 kg using a precision scale. Height measurement was done against a vertical wooden traditional height gauge with a horizontal pointer (Robé Medical, Remiremont, Grand-Est, France). To obtain precise measurement, standing straight patients were heighted without shoes and with feet together.

DXA: The fan-beam dual-energy X-ray absorptiometer (DXA) scanner (QDR 4500A; Hologic, Bedford, MA, USA) was used to assess whole body composition. During the waiting time before transplantation, measurements were performed on the day after dialysis using the weight measured on the same day. An empty peritoneal cavity was required for patients on peritoneal dialysis. Patient positioned was standardised. The DXA scans were acquired with the APEX 2 version 8.26 for QDR 4500A software version (classic calibration). Before 2009, the classic calibration method was used for the body composition (fat and lean soft tissue) calculation [10]. After 2009, a new software was developed with an upgrading of the APEX 2 version: the NHANES calibration provides a slightly different approach for body composition calibration [11]. The scans acquired after 2009 were re-analysed using the first version to ensure consistency of data and avoid measurement bias.

The values of whole-body composition are expressed as fat mass (FM) and fat-free mass (FFM) in absolute values, and as fat mass index (FMi, kg/m^2^) [FM indexed for height squared] and fat-free mass index (FFMi, kg/m^2^) [FFM and indexed for height squared].

Bioimpedance spectroscopy: Whole-body bioimpedance spectroscopy (BIS) was performed using a multifrequency device (Imp SFB7; ImpediMed, Pinkenba, QLD, Australia). Measurements were performed just after DXA evaluation, with a 10-min rest in the supine position. The electrodes and recording pads were placed on the non-assessment side of the body in patients with an arm access. In those with a central catheter or for patients on peritoneal dialysis, the dominant arm was chosen. The device scans 256 frequencies between 4 kHz and 1000 kHz for estimation of body composition, and it utilises Cole modelling with Hanai mixture theory to determine total body water (TBW), extracellular water (ECW) and intracellular water (ICW) [12].

BCM is defined as follows:

$$ \mathrm{BCM}\ \left(\mathrm{kg}\right)=\mathrm{FFM}\ {\left(\mathrm{kg}\right)}_{\mathrm{DXA}}-\mathrm{ECW}{\left(\mathrm{l}\right)}_{\mathrm{BIS}} $$

where DXA and BIS are performed on the same day.

### Physical activity level

Physical activity was estimated using the French version of the Baecke Physical Activity questionnaire [13, 14]. The self-perception of duration and intensity of three components of physical activity (work, leisure time and sport). leads to the calculation of scores. The sum of the scores obtained for these components leads to the global physical activity level for each patient.

### Statistical analysis

Statistical analysis was performed using SAS (ver. 9.2; SAS Institute Inc., Cary, NC, USA). The results are expressed as the median [interquartile range] for continuous variables and frequency for categorical variables. Patient characteristics at the time of listing (inclusion) were compared between transplanted and non-transplanted patients by Wilcoxon’s test for continuous variables and the χ^2^ test or exact Fisher’s test for categorical variables. A linear mixed regression model, with a random intercept and random slopes before and 1 month after KT, was used to examine the factors related to changes in BCM. The effects of each variable of interest were tested on the intercept (difference at KT) and each slope. Univariable analyses took into account the effects of only one variable; while multivariable analyses took into account all variables in the same model.

## Results

### Study population

**Fig. 2:**
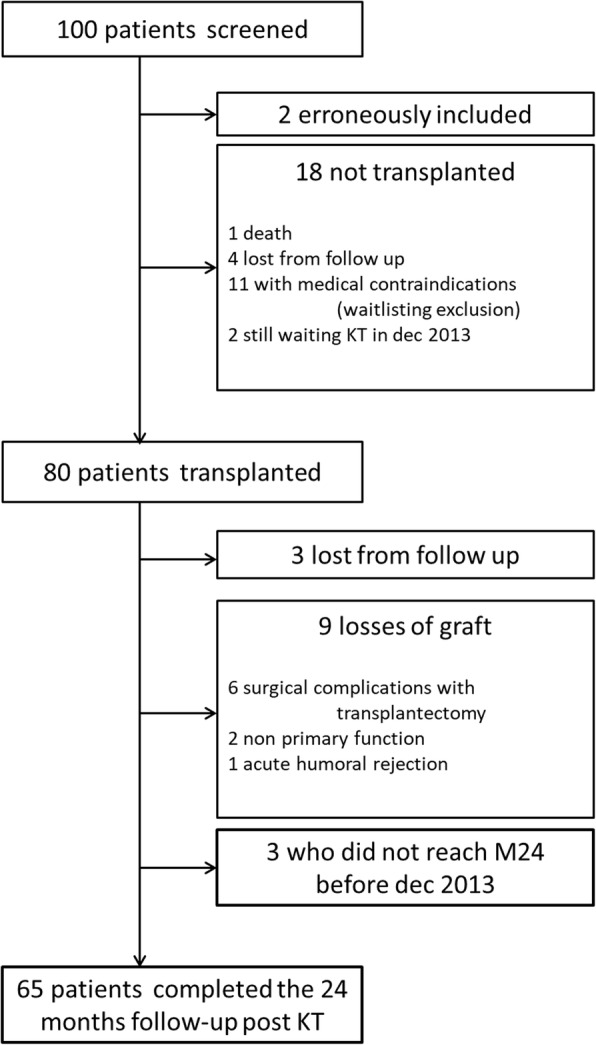
flow chart

One hundred consecutive candidates for kidney transplantation at the Renal Transplant Unit of Pellegrin University Hospital (Bordeaux, France) between August 2007 and January 2010 who met the inclusion criteria were enrolled in this study. Among them, two patients older than 66 years were not included in the analysis due to the exclusion criteria. The characteristics of the overall 98 patients included in the study were described previously [9]. Among them, 18 did not undergo transplantation (Fig. 2). For this group of patients, only five and two measurements were available at 12 and 24 months after listing, respectively. The two groups of patients (transplanted/not transplanted) were similar with regard to baseline characteristics (Table 1) with the exception of dialysis vintage at the time of inclusion (longer in the not transplanted patients). Eighty patients were finally transplanted after a median period of 433 [246–739] days. All data were analysed until exclusion (loss of graft or loss to follow-up). Only 65 patients completed the study and were analysed for the overall period (Fig. 2). Induction immunosuppressive therapy consisted of monoclonal anti-CD25 antibody in 80% of the patients (*n* = 47). Tacrolimus was used in all patients at the time of grafting.

**Table 1:** Patients characteristics at the time of listing (inclusion) compared to patients no transplanted

	Transplanted *n* = 80	Non transplanted *n* = 18	p
Age (years)	55.3 [46.0–60.6]	54.0 [49.5–58.1]	0.93
Gender (% male)	68.8	83.3	0.22
Initial nephropathy (%)			0.36
Glomerular	33.8	38.9	
Polycystic disease	22.5	16.7	
Tubulo-interstitial	15.1	22.3	
Others	28.6	22.1	
HD/PD (%)	93.8 / 6.3	88.9 / 11.1	0.30
Dialysis vintage (months)	7.0 [5.0–11.5]	11.5 [7.0–18.0]	0.03
BMI at inclusion (kg/m^2^)	25.2 [23.0–28.2]	27.6 [24.2–29.1]	0.34
BMI 12 months after inclusion (kg/m^2^)	25.5 [23.1–28.9]	27.6 [23.4–28.3]	0.99
BCM at inclusion (kg)	33.6 [27.8–38.7]	34.5 [32.8–40.1]	0.31
BCM 12 months after inclusion (kg)	32.0 [26.1–40.4]	32.8 [32.3–34.0]	0.92
Physical activity score at inclusion	6.4 [4.5–8.1]	6.4 [5.7–7.3]	0.92
Diabetes mellitus (%)	16.3	33.3	0.11

Median (interquartile ranges), %; *BMI* body mass index; *HD* haemodialysis; *PD* peritoneal dialysis; *BCM* Body Cell Mass

### Biochemical analysis/dietary recall

With the exception of patients with surgical complications or with non-primary function, creatinine levels decreased promptly after KT to reach 1.7 [1.4–2.2] mg/dL at 1 month and the creatinine nadir was observed at 12 months (1.4 [1.2–1.8] mg/dL) (Table 2). Nutritional biological parameters remained stable during the waiting period and after KT, despite low energy intake as determined by dietary recall (between 23 and 25 kcal/kg/d). Protein intake did not change throughout the study period. The results are presented in Table 2.

**Table 2:** nutritional parameters (median, min-max)

	Before KT		After KT					
	I	I + 12	Day 0	Month 1	Month 3	Month 6	Month 12	Month 24
Weight (kg)	71.5	72.5	73	70	70.8	72.7	74.5	75
min-max	44.0–83.9	66.0–82.0	65.0–83.0	61.0–79.0	61.0–81.1	61.5–81.5	63.0–82.5	64.0–85.0
n	80	39	78	74	72	71	68	65
BMI (kg/m2)	25.2	25.5	25.9	24.9	24.6	25.3	25.8	25.4
min-max	23.0–28.2	23.1–28.9	23.0–27.9	22.2–27.1	22.6–27.6	22.8–28.1	22.9–28.9	23.2–29.4
n	80	39	78	74	72	71	68	65
FMi (kg/m2)	8.6	7.8	/	7.5	/	7.4	8.7	8.5
min-max	6.2–10.4	6.2–10.9	/	5.6–9.4	/	5.7–9.3	6.8–10.1	6.8–10.1
n	78	37	/	44	/	46	66	63
FFMi (kg/m2)	16.8	16.9	/	15.9	/	16.6	16.8	16.6
min-max	14.8–18.3	15.1–18.7	/	14.5–16.9	/	15.2–18.2	15.1–18.5	15.3–18.6
n	78	37	/	44	/	46	66	63
TBW (l) min-max	36.7 30.6–42.4	39.9 33.0–45.0	/	36.7 32.7–42.7	/	38.4 34.4–44.3	39.3 34.3–46.4	40.7 33.3–46.6
n	79	38	/	70	/	69	66	62
ECW (l)	14.8	15.8	/	15.8	/	16.2	16.4	17.1
min-max	11.7–17.2	13.2–17.5	/	13.8–18.0	/	14.0–19.0	14.0–19.0	14.9–19.7
n	79	38	/	70	/	69	66	62
BCM (kg)	33.6	32	/	30.4	/	31.5	32.3	30.9
min-max	27.8–38.7	26.1–40.4	/	25.6–34.0	/	25.5–35.7	26.1–35.5	25.5–36.2
n	78	37	/	44	/	46	66	61
Protein (g/Ll)	73	76	75	70	70	70	71	70
min-max	68–79	63–79	70–80	65–73	66–73	67–74	65–76	66–74
n	80	39	79	75	72	71	68	65
Albumin (g/l)	46	45	44	43	44	45	43	43
min-max	43–49	43–47	41–47	40–46	41–47	41–47	41–46	40–45
n	77	39	77	48	45	47	65	65
Pre albumin (g/l)	0.4	0.4	0.4	0.3	0.3	0.3	0.3	0.3
min-max	0.4–0.5	0.4–0.5	0.3–0.5	0.3–0.4	0.3–0.3	0.3–0.3	0.3–0.3	0.3–0.4
n	75	37	70	47	44	46	61	64
CRP (mg/l)	2	2	3	5	3	4	4	3
min-max	1.0–8.0	1.0–5.0	1.5–6.5	2.0–13.0	1.0–7.0	2.0–7.0	2.0–7.0	1.0–6.0
n	79	37	56	46	38	57	66	65
Creatinine (mg/dL)	7.84	8.23	7.77	1.70	1.53	1.54	1.42	1.50
min-max	6.15–9.34	6.39–10.40	6.12–9.85	1.37–2.20	1.32–1.87	1.28–1.84	1.22–1.83	1.06–1.81
n	80	39	79	75	72	71	68	65
Energy intake (Kcal/kg/d)	23.4	22.4	/	23.8	23.0	24.8	24.9	23.4
min-max	19.5–28.3	18.9–27.7	/	17.6–28.4	19.5–28.1	20.9–29.2	19.7–28.8	20.2–29.8
n	64	27	/	53	49	45	43	38
Protein intake (g/kg/d)	1.1	0.9	/	1.1	1.1	1.1	1.0	1.1
min-max	0.9–1.2	0.8–1.2	/	0.8–1.5	0.9–1.2	0.9–1.3	0.8–1.3	0.9–1.3
n	64	27	/	53	49	45	43	38
PA score	6.4	5.6	/	4.4	/	5.5	6.2	5.9
n	4.5 6 8.1 78	4.5–7.2 36	/	3.7–5.5 67	/	4.4–6.8 68	4.7–7.4 66	4.9–7.4 62

*BMI* Body Mass Index; *FMi* Fat Mass index; *FFMi* Fat Free Mass index; *TBW* Total Body Water, *ECW* Extra Cellular Water; *BCM* Body Cell Mass; *CRP* C Reactive Protein; *PA* Physical Activity

### Whole-body composition

#### Whole-body weight

In the first weeks post-KT, body weight decreased, reaching a nadir at 1 month (70 [61–79] kg). It then increased until 12 months to reach 74.5 [63.0–82.5] kg and then remained stable (75.0 [64.0–85.0 kg] up to 24 months. This evolution was more pronounced in men. According to the WHO criteria, at the time of inclusion, 3.8% of our patients could be classified as lean (BMI < 18.5 kg/m^2^), 38.8% as overweight (25 < BMI < 30) and 12.5% as obese (BMI > 30). At the time of transplantation, 3.8% were lean, 47.4% were overweight and 11.5% were obese. Two years post-KT, the proportions of overweight and obese patients increased (4.6% were lean, 33.8% were overweight and 21.5% were obese).

#### Body compartments (Table 2)

**Fig. 3:**
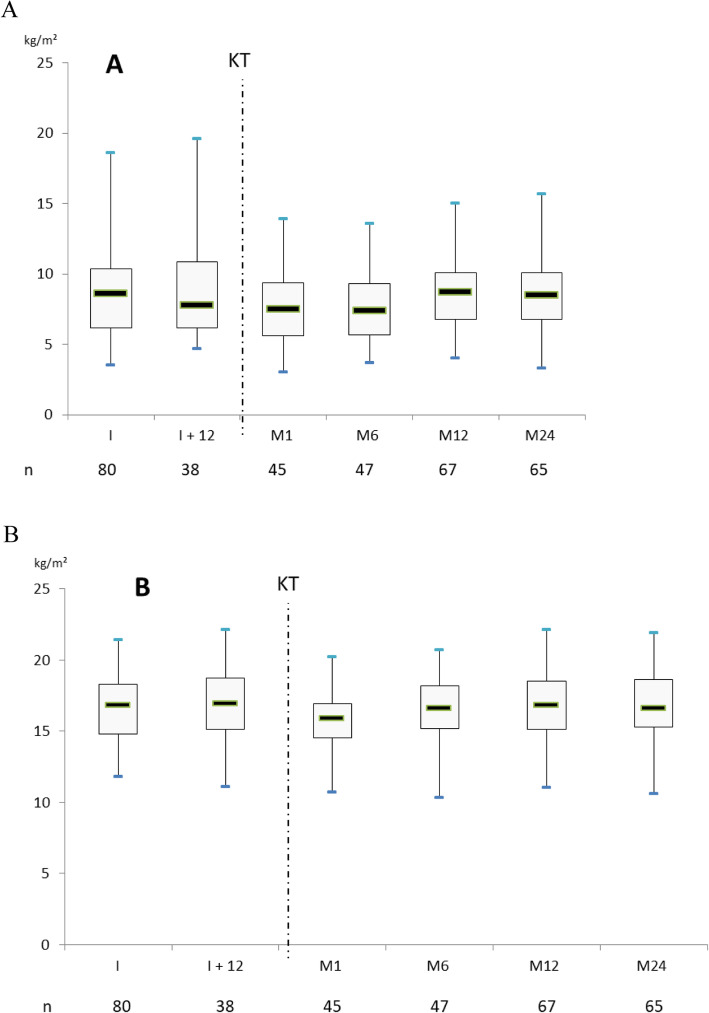
**a** Fat Mass index (FMi) evolution before and after kidney transplantation (KT). Measurements were done at the time of listing (I), 12 months later if not transplanted (I +  12) and fter KT at month 1 (M1), 6 (M6), 12 (M12) and 24 (M24). **b** Fat Free Mass index (FFMi) evolution before and after kidney transplantation (KT). Measurements were done at the time of listing (I), 12 months later if not transplanted (I +  12) and after KT at month 1 (M1), 6 (M6), 12 (M12) and 24 (M24)

**Fig. 4:**
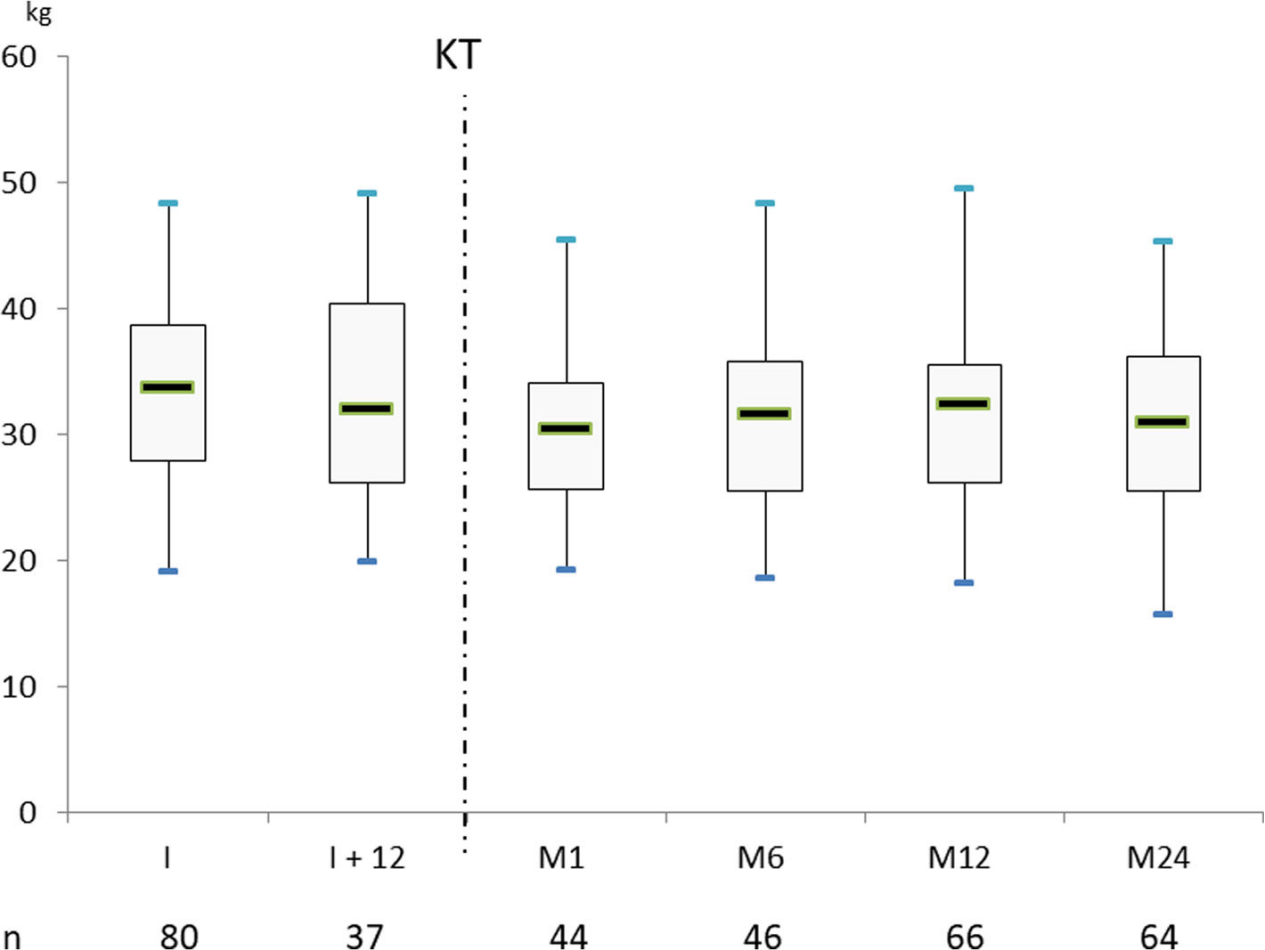
Body cell mass (BCM) evolution before and after kidney transplantation (KT). Measurements were done at the time of listing (I), 12 months later if not transplanted (I +  12) and after KT at month 1 (M1), 6 (M6), 12 (M12) and 24 (M24)

After transplantation, the nadirs of BCM and FFMi were observed at 1 month (30.4 [25.6–34] kg and 15.9 [14.5–16.9] kg/m^2^, respectively). BCM and FFMi reached their maximum levels at 12 months (32.3 [26.1–35.5] kg and 16.8 [15.1–18.5] kg/m^2^, respectively) and were similar to the last levels observed before transplantation. Over the next 12 months, BCM and FFMi decreased slightly. FMi also decreased after kidney transplantation, but the nadir was measured at 6 months after KT (7.4 [5.7–9.3] kg/m^2^) and then increased rapidly until 12 months (8.7 [6.8–10.1] kg/m^2^), remaining stable thereafter (Figs. 3a, b and 4).

### Physical activity

The global physical activity level decreased slightly during the waiting period before KT and the nadir was observed at 1 month post-KT. One year after KT, physical activity returned to the previous level at the time of listing (Table 2). Analysis of the three components of physical activity indicated that the main changes were due to recovery of professional-related physical activity.

### Predictors of BCM changes

We evaluated the factors related to changes in BCM (Table 3). In univariable analysis, several factors, i.e. sex, albumin level, energy and protein intake, physical activity level and corticosteroid dose, were associated with changes in BCM. In multivariable analysis, age and recipient’s sex, total physical activity level and daily corticosteroid dose had significant independent effects on BCM; male recipient, higher physical activity level and lower corticosteroid dose were associated with better BCM recovery after KT.

**Table 3:** Predictors of Body Cell Mass (BCM) changes

		Univariable analysis			Multivariable analysis		
	n (observations)	β	IC _95%_ β	p	β	IC _95%_ β	p
Age (+ unit)	80 (340)	−0.08	[−0.23;0.06]	0.26	−0.12	[−0.23;-0.02]	0.02
Gender (Female vs Male)	80 (340)	−11.29	[−13.62;-8.96]	< 10^−4^	−11.62	[−13.93;-9.30]	< 10^−4^
Dialysis vintage (+ 1 unit)	80 (340)	− 0.09	[− 0.22;0.03]	0.15	− 0.06	[− 0.14;0.02]	0.14
Albumin (+ 1unit)	78 (335)	0.11	[0.04;0.18]	< 10^−2^	0.02	[−0.06;0.10]	0.62
CRP (+ 1 unit)	79 (333)	−0.03	[−0.06;0.01]	0.09	−0.03	[− 0.07;0.01]	0.13
Energy intake (+ 1 unit)	69 (243)	−0.10	[−0.16;-0.03]	< 10^−2^	− 0.06	[− 0.15;0.03]	0.17
Protein intake (+ 1 unit)	69 (243)	−1.93	[−3.47;-0.40]	0.01	−1.05	[−3.12;1.02]	0.31
Physical activity level (+ 1 unit)	75 (329)	0.41	[0.20;0.61]	10^−4^	0.34	[0.11;0.57]	< 0.01
Corticosteroid dose (+ 1 unit)	75 (326)	−0.05	[−0.07;-0.03]	< 10^−4^	− 0.04	[− 0.06;-0.02]	< 0.01

## Discussion

The present longitudinal study confirmed that the whole-body composition is significantly modified by KT. Most of the changes appear in the first 12 months and compensate for weight loss due to LBM and BCM losses occurring before KT. These changes are related to KT factors (corticosteroid therapy) and patient behaviour (physical activity).

Successful KT is associated with prompt changes in weight and body composition. Previous studies have demonstrated that weight increases in the few weeks after KT and that this is mostly related to gain in fat mass [3, 15, 16]. Weight gain after KT is considered a risk factor for both short- and long-term graft and patient survival [5, 8, 17]. However, in these two retrospective studies, weight gain was defined according to BMI evolution and no data regarding body composition (with a focus on LBM) were available. BMI does not differentiate between muscle and fat tissue. The data from our study population published previously demonstrated that despite good nutritional status and normal BMI, the patients could be considered as having been sarcopenic before transplantation [9]. In both haemodialysis and peritoneal dialysis, several studies have suggested that muscle mass rather than BMI is the key to the obesity paradox in end-stage renal disease [18]. In kidney transplant candidates, a higher muscle mass is associated with higher rates of post-transplant patient and graft survival [19]. Only one study linked body composition to mortality after kidney transplantation; higher risk was observed in patients with higher visceral adiposity estimated by waist circumference [20]. These data extend the importance of assessment of muscle mass and of developing strategies in patients before and after kidney transplantation to preserve/promote gain of muscle mass after KT.

In this context, the relevance of the body composition assessment tools in cases of renal failure must be raised. The key to assessment of nutritional status is the ability to perform serial measurements over time to detect changes in overhydrated patients. In a 4 year-longitudinal study, it was not possible to maintain a constant hydration status in our population. To avoid this limitation, we studied the evolution of BCM by combination of the results of both DXA (with a low dose of radiation) and BIS (performed at bedside) [21]. Lean body mass (LBM) consists of extracellular mass (mainly ECW) and BCM, where BCM consists of all metabolically active cells of the body. Modification of LBM can be masked by modification of hydration status. BCM is a potentially sensitive indicator of muscle mass. It is also strongly correlated with handgrip strength in both healthy subjects and haemodialysis patients [22]. Therefore, it may be useful as a tool to evaluate both muscle mass and function in patients.

In our population, evolution of body weight seemed appropriate: patients returned to the observed pre-transplant weight with increases in both fat and muscle mass, with total BCM recovery. During the waiting period, we observed a decrease in BCM. LBM and BCM decrease after initiation of dialysis have been described previously [23, 24] and were shown to be influenced by the renal replacement treatment modality [25]. Thus, kidney transplantation-related body composition changes in this context can then be considered as beneficial. Weight gain after KT is multifactorial with non-modifiable factors (genetics, age, sex) and modifiable factors (socioeconomic status, physical level, dietary habits). This is crucial in the early post-transplant period with increased appetite. Furthermore, immunosuppressive medications can lead to alteration of muscle metabolism; steroids are associated with catabolic effects [26] and calcineurin inhibitors with skeletal muscle abnormalities [27, 28]. Most of these classical risk factors were associated in our population with whole-body composition changes: sex, albumin level, energy and protein intake, physical activity level and corticosteroid dose were associated with changes in BCM. Male recipients, higher level of physical activity and lower corticosteroid dose were still significant in adjusted analysis of post-KT BCM recovery. The role of physical activity in body composition in KTR is still a matter of discussion. In a French transversal study, weight gain post KT was higher in patients with low physical activity compared to patients with a higher physical activity level [29]. In a longitudinal study in 26 patients, patients with low physical activity had fat mass gain during the first year post-KT, whereas fat mass remained stable in patients with high a level of physical activity [16]. No data were available about fat-free mass in this population. The influence of corticosteroid maintenance after KT has been well described—weight gain after the first year post-KT is significantly and positively related to cumulative steroid dose [30], and late steroid withdrawal (after 1 year) leads to a slight but significant loss of body weight [31]. Finally, El-Haggan et al. demonstrated that corticosteroid withdrawal leads to gain in fat-free mass, whereas steroid maintenance is associated with fat mass gain. In the two groups, weight was not different at the end of the 2-year period of the study, underlining the importance of body-composition monitoring [32].

Our study had several major strengths. First, this was a longitudinal, prospective study with a start before KT, while previous studies focused only on post-KT outcomes. Second, whole-body composition and physical activity levels were assessed using strong validated tools.

However, this study also had some limitations. First, protein and energy intakes were estimated using a 3-day food record. Based on this method, energy intake was about 25 kcal/kg/d in our population regardless of the period considered. However, this was not consistent with the weight and BCM gains after KT, suggesting large underreporting of energy intake, which is consistent with published data [33]. Similarly, protein intake was estimated as 1.1 g/kg/d but was not compared to the 24-h urinary urea nitrogen measurement. Second, muscle function was not estimated directly. We assessed physical activity by questionnaire, which may have diluted the effect of this variable, although it was statistically significant. Finally, although this is one of the largest studies performed to date, the number of patients did not allow us to test the specific effects of sex or therapeutic classes of immunosuppressive treatment on the BCM evolution post-KT.

## Conclusion

This longitudinal study confirmed that successful KT is associated with whole-body composition modifications. The results showed that weight gain after KT can be associated with safe BCM recovery, which is strongly influenced by sex, corticosteroid therapy and physical activity level. In the modern area of KT, avoidance of long-term metabolic complications is a therapeutic challenge. All of the strategies based on safe lifestyle behaviours should be developed in this population, before and after KT. Promotion of physical activity and a healthy diet together with management of immunosuppressive therapy may have beneficial effects in terms of the prevention of metabolic complications in KTR.

## Data Availability

The datasets used and analyzed during the current study are available from the corresponding author on reasonable request.

## Abbreviations

BCM: Body Cell Mass
BMI: Body Mass Index
BIS: Bio impedancemetry spectroscopic
DXA: Dual X-ray absorptiometry
ECW: Extra Cellular Water
FFM: Fat Free Mass
FFMI: Fat Free Mass Index
FM: Fat Mass
FMI: Fat Mass Index
HD: Haemodialysis
ICW: Intra Cellular Water
LBM: Lean Body Mass
PA: Physical activity
PD: Peritoneal dialysis
TBW: Total Body Water
KT: Kidney transplantation
KTR: kidney transplant recipient
