# Correction to: Evolution of body composition following successful kidney transplantation is strongly influenced by physical activity: results of the CORPOS study

**DOI:** 10.1186/s12882-021-02258-5

**Published:** 2021-02-16

**Authors:** Karine Moreau, Aurélie Desseix, Christine Germain, Pierre Merville, Lionel Couzi, Rodolphe Thiébaut, Philippe Chauveau

**Affiliations:** 1 Renal Transplant Unit, Pellegrin Hospital, Bordeaux, France; 2 Clinical Epidemiology Unit, Bordeaux University Hospital, Bordeaux, France; 3 CNRS-UMR 5164 immunoConcEpT Bordeaux University, Bordeaux, France; 4 INSERM U1219 Bordeaux Population Health, Bordeaux, France; 5 Bordeaux University, Bordeaux, France; 6 AURAD Aquitaine, Gradignan, France

**Correction to: BMC Nephrol 22, 31 (2021)**

**https://doi.org/10.1186/s12882-020-02214-9**

Following publication of the original article [1], we have been informed that the authors were incorrectly affiliated. The author group has been updated above with the correct affiliation.

The original article has been corrected.
